# Minocycline attenuates lipopolysaccharide (LPS)-induced neuroinflammation, sickness behavior, and anhedonia

**DOI:** 10.1186/1742-2094-5-15

**Published:** 2008-05-13

**Authors:** Christopher J Henry, Yan Huang, Angela Wynne, Mark Hanke, Justin Himler, Michael T Bailey, John F Sheridan, Jonathan P Godbout

**Affiliations:** 1 Department of Molecular Virology, Immunology and Medical Genetics, The Ohio State University, 333 W. 10th Ave, Columbus, OH 43210, USA; 2 Department of Oral Biology, The Ohio State University, 305 W. 12th Ave, Columbus, OH 43210, USA; 3 Institute for Behavioral Medicine Research, The Ohio State University, 333 W. 10th Ave, Columbus, OH 43210, USA

## Abstract

**Background:**

Activation of the peripheral innate immune system stimulates the secretion of CNS cytokines that modulate the behavioral symptoms of sickness. Excessive production of cytokines by microglia, however, may cause long-lasting behavioral and cognitive complications. The purpose of this study was to determine if minocycline, an anti-inflammatory agent and purported microglial inhibitor, attenuates lipopolysaccharide (LPS)-induced neuroinflammation, sickness behavior, and anhedonia.

**Methods:**

In the first set of experiments the effect of minocycline pretreatment on LPS-induced microglia activation was assessed in BV-2 microglia cell cultures. In the second study, adult (3–6 m) BALB/c mice received an intraperitoneal (i.p.) injection of vehicle or minocycline (50 mg/kg) for three consecutive days. On the third day, mice were also injected (i.p.) with saline or *Escherichia coli *LPS (0.33 mg/kg) and behavior (i.e., sickness and anhedonia) and markers of neuroinflammation (i.e., microglia activation and inflammatory cytokines) were determined. In the final study, adult and aged BALB/c mice were treated with the same minocycline and LPS injection regimen and markers of neuroinflammation were determined. All data were analyzed using Statistical Analysis Systems General Linear Model procedures and were subjected to one-, two-, or three-way ANOVA to determine significant main effects and interactions.

**Results:**

Minocycline blocked LPS-stimulated inflammatory cytokine secretion in the BV-2 microglia-derived cell line and reduced LPS-induced Toll-like-receptor-2 (TLR2) surface expression on brain microglia. Moreover, minocycline facilitated the recovery from sickness behavior (i.e., anorexia, weight loss, and social withdrawal) and prevented anhedonia in adult mice challenged with LPS. Furthermore, the minocycline associated recovery from LPS-induced sickness behavior was paralleled by reduced mRNA levels of Interleukin (IL)-1β, IL-6, and indoleamine 2, 3 dioxygenase (IDO) in the cortex and hippocampus. Finally, in aged mice, where exaggerated neuroinflammation was elicited by LPS, minocycline pretreatment was still effective in markedly reducing mRNA levels of IL-1β, TLR2 and IDO in the hippocampus.

**Conclusion:**

These data indicate that minocycline mitigates neuroinflammation in the adult and aged brain and modulates the cytokine-associated changes in motivation and behavior.

## Background

The bi-directional communication between the immune system and the central nervous system (CNS) is necessary for mounting the appropriate immunological, physiological, and behavioral responses to immune stimulation [1]. CNS innate immune cells including microglia and macrophages play integral roles in receiving and propagating inflammatory signals that are initiated at the periphery. Activation of peripheral innate immune cells elicits the secretion of inflammatory cytokines, including interleukin (IL)-1, IL-6, and tumor necrosis factor-α (TNFα.), that use neural [2,3], humoral [4] and blood brain barrier pathways [5] to relay this signal to the CNS. This inflammatory signal, in turn, induces CNS macrophages and microglia to produce the same cytokines [6], which target neuronal substrates and elicit a sickness behavior syndrome that is normally adaptive and beneficial to the host [1]. An amplified or excessive inflammatory cytokine response in the brain, however, is associated with a myriad of complications including cognitive dysfunction [7-10], prolonged sickness behavior [11-14], and depressive-like behavior [15].

Microglia are primarily involved in immune surveillance [16,17], but when activated have macrophage-like capabilities including phagocytosis, inflammatory cytokine production, and antigen presentation [18]. Normally these neuroinflammatory changes are transient with microglia returning to a resting state as the immune stimulus is resolved. Aging or neurological disease, however, may provide a brain environment where microglia are more "reactive or primed" to a peripheral immune challenge [19]. Recent findings indicate that several markers of glial activation such as major histocompatibility complex (MHC) class II, complement receptors, and scavenger receptors are increased in brain during normal aging [13,20-26]. Furthermore, we and others have reported that a biological consequence of this reactive glial profile is an exaggerated neuroinflammatory response to innate immune challenge [9,10,12-14,27,28].

Active microglia and CNS macrophages also contribute to the production of oxidative and neuroactive mediators that may influence behavior. For instance, inflammatory cytokines in the CNS upregulate the enzyme IDO [29,30], which metabolizes tryptophan (TRP) into L-kynurenine (KYN) [31]. TRP degradation to KYN can reduce TRP levels that are required for serotonin synthesis [32] and can lead to the production of neuroactive mediators including 3-hydroxykynurenine (3HK) and quinolinic acid (QUIN) [31]. High levels of 3HK and QUIN induce neuronal damage through oxidative stress [33] and over stimulation of N-methyl-D-aspartate (NMDA) receptors [34,35]. A recent study indicates that while several cell types in the CNS express IDO, only microglia maintain all the enzymes required to produce 3HK and QUIN [36]. Because IDO mediated TRP degradation impacts both serotonergic and glutamatergic pathways, this may be an important mechanism underlying mood and behavior complications concomitant with inflammation [37-39].

Because activated microglia are suspected to cause or exacerbate several neurodegenerative diseases, pharmacological strategies to suppress microglial activity are being explored as therapies. Minocycline is a tetracycline derived antibiotic that has anti-inflammatory properties in the CNS that are separate from its antimicrobial action [40]. Minocycline readily crosses the blood brain barrier and attenuates inflammation associated with microglial activation. For example, minocycline blocks the deleterious effects of neuroinflammation on neurogenesis, long-term potentiation, and neuronal survival [41-43]. The mechanism of action is unclear, but recent studies indicate that minocycline abrogates MAPkinase and NFκB dependent signaling pathways in primary microglia and microglia cell cultures [44]. Moreover, in the brain of rats, minocycline abrogates microglial expression of CD11b and MHC II through a protein kinase-c dependent mechanism [45]. This is relevant because minocycline attenuates neuroinflammation in several rodent models of disease including Amyotrophic Lateral Sclerosis [46], Experimental Autoimmune Encephalomyelitis (EAE) [45] and MPTP-induced Parkinson's disease [47]. However, the extent to which minocycline facilitates the recovery from cytokine-mediated sickness behavior is unknown.

The present study investigated the degree to which minocycline–an anti-inflammatory agent and purported microglial inhibitor–reduced LPS-induced neuroinflammation and sickness behavior. We show that minocycline blocked LPS-stimulated inflammatory cytokine secretion in the BV-2 microglia-derived cell line and reduced LPS-induced Toll-like-receptor-2 (TLR2) surface expression on brain microglia. Moreover, our data show that minocycline pretreatment attenuated LPS-induced weight loss, social withdrawal, and anhedonia in adult mice. The attenuation of sickness behavior was paralleled with minocycline dependent decrease in markers of neuroinflammation (IL-1β, TLR2, and IDO) in adult and aged mice. These findings support our hypothesis that the ability to mitigate cytokine expression in the brain during systemic inflammatory events may be useful in preventing cognitive and behavioral deficits.

## Methods

### Animals

Male BALB/c mice, adults (3 month old) and juvenile (3–4 week old) were purchased from Harlan (Indianapolis, IN). For age comparisons, male BALB/c mice (3–4 and 20–22 month old) were purchased from the National Institute on Aging specific pathogen free colony. Upon arrival, mice were individually housed in polypropylene cages and maintained at 21°C under a 12 h light: 12 h dark cycle with *ad libitum *access to water and rodent chow. At the end of each study, mice were examined postmortem for gross signs of disease (e.g., splenomeglia or tumors). Data from mice determined to be unhealthy were excluded from analysis (< 5%). All procedures were in accordance with the National Institute of Health Guidelines for the Care and Use of Laboratory Animals and were approved by The Ohio State University Institutional Laboratory Animal Care and Use Committee.

### Cell culture

BV-2 microglia cell lines were cultured in growth medium (DMEM supplemented with 10% FBS, sodium bicarbonate 3.7 g/l, 200 mM glutamine, 100 U/ml penicillin G, 100 μg/ml streptomycin, 0.25 μg/ml fungizone) as previously described [12]. Cultures were maintained at 37°C with 95% humidity and 5% CO_2 _and growth medium was replenished every third day until confluence. Cultures were washed twice and supplemented with warm growth medium containing experimental treatments. Cell viability was measured by the MTS cell proliferation assay according to the manufacturer's instructions (Promega, Madison, WI).

### CNS macrophage/microglia isolation

CNS macrophages and microglia were collected from whole brain homogenates as described previously [48], but with several modifications. Mice were euthanized by CO_2 _asphyxiation and whole brains were collected. Brains were homogenized in Hank's balanced salt solution (HBSS) pH 7.4. Brain homogenates were passed through a 70 μm nylon cell strainer and centrifuged at 400 × g for 10 min. Supernatants were removed and cell pellets were re-suspended in 70% isotonic Percoll (GE-healthcare, Uppsala, Sweden) at room temperature. A discontinuous Percoll density gradient was set up as follows: 70%, 35%, and 0% isotonic Percoll. This suspension was centrifuged for 30 minutes at 400 × g. A mixed population of CNS macrophages and microglia was collected from the interphase between the 70% and 35% Percoll layers. Cells were washed and then re-suspended in sterile HBSS. The number of viable cells was determined using a hemacytometer and 0.2% trypan blue staining.

### Flow cytometry

Flow cytometric analysis of microglial cell surface markers was performed as described previously, but with a few modifications [48]. In brief, Fc receptors on macrophages and microglia were blocked with anti-CD16/CD32 antibody (eBiosciences, CA). Next, cells were incubated with either Panel-1 (anti-CD11b APC, anti-CD45 FITC, and anti-MHC II PE from eBiosciences, CA) or Panel-2 antibodies (anti-CD11b APC, anti-CD45 FITC, and anti-TLR2 PE from eBiosciences, CA). Expression of these surface receptors was determined by flow cytometry using a Becton-Dickinson FACSCaliber four color Cytometer. Thirty thousand events were collected and microglia were differentiated from macrophages based on the levels of CD11b and CD45 surface expression. Microglia stain CD11b^+^/CD45^low ^and macrophages stain CD11b^+^/CD45^high ^[48,49]. Flow data were analyzed using FlowJo software (Tree Star, San Carlos, CA).

### Behavior tests

#### Social exploratory behavior

To assess the motivation to engage in social exploratory behavior, a novel juvenile conspecific was introduced into the test subject's home cage for a 10-min period. Behavior was video taped and the cumulative amount of time the subject engaged in social investigation was determined from the video records by a trained observer who was blind to the experimental treatments. Baseline social behavior was measured at time 0 for all experimental treatments. Social behavior was determined as the amount of time that the experimental subject spent investigating (e.g., anogenital sniffing, trailing) the juvenile. Results are expressed as percent decrease in time engaged in social behavior compared to respective baseline measures.

#### Sucrose preference

To assess sucrose preference, mice were provided two solutions, water or water supplemented with 2% sucrose, in 50 ml conical tubes with stoppers fitted with ball-type sipper tubes. Prior to testing conditions, all mice were acclimated to the two bottle test choice. All mice drank both the water and the 2% sucrose solution, but preferred drinking the sucrose over the water (data not shown). On the day of testing, mice were fluid and food deprived for 2 h prior to testing [50]. At the start of the dark phase of the photoperiod, drinking water and the 2% sucrose solution were placed in the home cage overnight (15 h). At the end of each testing period the fluid content of the conical tubes was measured and sucrose preference was determined using the equation: Sucrose intake/Total fluid intake (water + sucrose intake) × 100 [51].

### Plasma cytokine measurement

IL-6 and IL-1β were measured in the plasma as previously described [52]. In brief, mice were euthanized by CO_2 _asphyxiation and blood was collected by cardiac puncture into EDTA coated syringes. Samples were centrifuged (6000 × g for 15 min at 4°C) and plasma was collected and stored frozen (-80°C) until assaying. Plasma samples were assayed for IL-6 using a customized ELISA that we have described in detail [52] and for IL-1β using a commercial ELISA kit (R&D Systems, Minneapolis, MN). Assays were sensitive to 8 pg/ml of IL-6 and 1.5 pg/ml of IL-1β, and inter- and intra-assay coefficients of variation were less than 10%.

### Real time PCR

Total RNA was isolated from brain using the Tri Reagent protocol (Sigma, St. Louis, MO). RNA samples were subjected to a DNase I digestion procedure and then reverse transcribed to cDNA using a RT RETROscript kit (Ambion, Austin, TX). Quantitative real time PCR was performed using the Applied Biosystems (Foster, CA) Assay-on Demand Gene Expression protocol as previously described [13]. In brief, cDNA was amplified by real time PCR where a target cDNA (IL-1β, IL-6, MHC II, TLR2, or IDO) and a reference cDNA (glyceraldehyde-3-phosphate dehydrogenase) were amplified simultaneously using an oligonucleotide probe with a 5' fluorescent reporter dye (6-FAM) and a 3' quencher dye (NFQ). Fluorescence was determined on an ABI PRISM 7300-sequence detection system (Applied Biosystems, CA). Data were analyzed using the comparative threshold cycle (Ct) method and results are expressed as fold difference.

### Experimental protocols

For the cell culture studies, minocycline was prepared in dimethyl sulfoxide (DMSO) and BV-2 cells were washed and replenished with growth mediumsupplemented with 0, 25, 50, 100, 200, or 400 μg/ml minocycline. After 30 min, LPS at 10 ng/ml was added to the culture medium. Supernatants were collected 4 h later and IL-6 and IL-1β concentrations were determined by ELISA. Total proteins were determined from cell culture homogenates by the Bio-Rad Dc protein assay according to the manufacturer's instructions (Bio-Rad Lboratories, Hercules, CA). Each treatment was replicated a minimum of four times. Cell viability was confirmed by the MTS cell proliferation assay according to the manufacturer's instructions (Promega, Madison, WI).

For all mouse studies, minocycline (Sigma, St. Louis, MO) was dissolved in sterile water and sonicated to ensure complete solubilization. In the first mouse study, adult male BALB/c mice received an intraperitoneal (i.p.) injection of vehicle or minocycline (50 mg/kg) for three consecutive days. On the 3^rd ^day, mice were also injected i.p. with saline or *Escherichia coli *LPS (0.33 mg/kg; serotype 0127:B8, Sigma, St. Louis, MO) and were euthanized by CO_2 _asphyxiation 24 h later (n = 6). The LPS dosage was selected because it elicits a proinflammatory cytokine response in the brain resulting in mild transient sickness behavior in adult mice [13,53]. Macrophage/microglial cells were isolated from whole brain homogenates and TLR2 and MHC II surface expression were determined by flow cytometry. The minocycline injection regimen and dosage was selected because a repeated pretreatment course with minocycline is necessary to attenuate neuroinflammation [41-43,45].

In the second study, adult male BALB/c mice received an i.p. injection with vehicle or minocycline for three consecutive days. On the third day, motivation to engage in social behavior was determined immediately before i.p. injection of saline or LPS (0.33 mg/kg) and again 2, 4, 8, 12, and 24 h later (n = 8). Body weight and food intake were measured at each time point over the 24 h period. In a related, but separate study, adult mice were treated with minocycline and LPS as described and anhedonia was assessed 24–39 h following i.p. injection of saline or LPS (0.33 mg/kg) (n = 15). Body weight, food intake, water intake, and sucrose intake were determined over the testing period.

In the third study, adult BALB/c mice were treated with minocycline and then LPS as described. Mice were euthanized by CO_2 _asphyxiation 4 later. Brains were removed and dissected to collect different brain regions. Brain regions were stored at -20°C in RNAlater (Qiagen, CA). Total RNA was isolated from brain samples and assayed using quantitative PCR (n = 8). Plasma was also collected and stored (-80°C) until assaying.

In a final study, adult (3–4 month old) or aged (20–22 month old) male BALB/c mice were treated with minocycline and LPS as described and euthanized 4 h later. Brains were dissected to collect different brain regions and were stored at -20°C in RNAlater (Qiagen, CA). Total RNA was isolated from the hippocampus and assayed using quantitative PCR (n = 8).

### Statistical analysis

All data were analyzed using Statistical Analysis Systems (SAS) General Linear Model procedures. Data were subjected to one, two- (Mino × LPS, Age × LPS, Mino × Age) or three-way (Mino × LPS × Time, Mino × LPS × Age) ANOVA to determine significant main effects and interactions between main factors. When appropriate, differences between treatment means were evaluated by an *F*-protected *t*-test using the Least-Significant Difference procedure of SAS. All data are expressed as treatment means ± standard error of the mean (SEM).

## Results

### Minocycline attenuates LPS-induced cytokine production in BV-2 microglia

**Figure 1:**
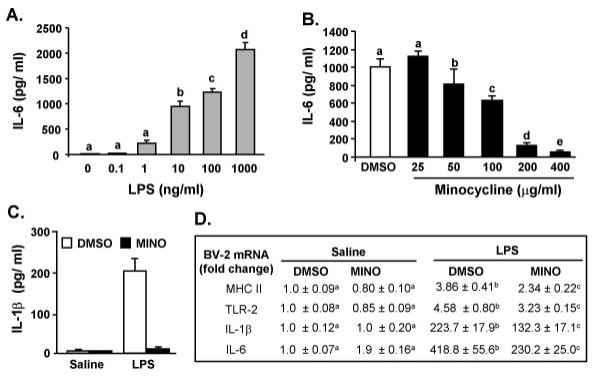
**Minocycline suppresses LPS-stimulated expression and production of cytokines in BV-2 microglia cultures**. A) BV-2 cells were stimulated with 0, 0.1, 1, 10, 100, or 1000 ng/ml LPS and IL-6 protein levels were determined from supernatants collected 4 h later. B) BV-2 cells were pretreated with 0, 25, 50, 100, 200, or 400 μg/ml minocycline for 30 min and then treated with LPS (10 ng/ml). IL-6 protein levels were determined from supernatants collected 4 h later. C) BV-2 cells were treated with 200 μg/ml minocycline for 30 min and then treated with 10 ng/ml LPS. IL-1β protein levels were determined from supernatants collected 4 h later. D) BV-2 cells were treated as above and MHC II, TLR2, IL-1β, and IL-6 mRNA levels were determined. For each cell culture experiment, results are an average of 4 independent experiments. Means with different letters (a, b, c, d, or e) are significantly different (*P *< 0.05) from each other.

Minocycline is a tetracycline-type antibiotic that has anti-inflammatory properties in the CNS [41-43,45]. To determine the degree to which minocycline suppresses microglia activation, BV-2 microglia-derived cell lines were used. In the first experiment, BV-2 cells were treated with LPS and IL-6 production was determined 4 h later. Fig. 1A shows that LPS increased IL-6 production in a dose dependent manner F(5, 23) = 101, *P *< 0.001). In the second experiment, BV-2 cells were incubated with DMSO or minocycline and then stimulated with LPS. Minocycline reduced LPS-induced IL-6 secretion in a dose dependent manner (Mino × LPS interaction, F(4, 23) = 16.87, *P *< 0.001, Fig. 1B). Minocycline pretreatment had a similar anti-inflammatory effect on LPS-stimulated IL-1β secretion (Fig. 1C). In a third experiment, minocycline suppressed LPS-induced MHC II, TLR2, IL-1β, and IL-6 mRNA levels (*P *< 0.05, for each, Fig. 1D). The MTS assay verified that neither cell survival nor proliferation was affected by the experimental treatments (data not shown).

### LPS-induced TLR2 surface expression on microglia is reduced by minocycline

**Figure 2:**
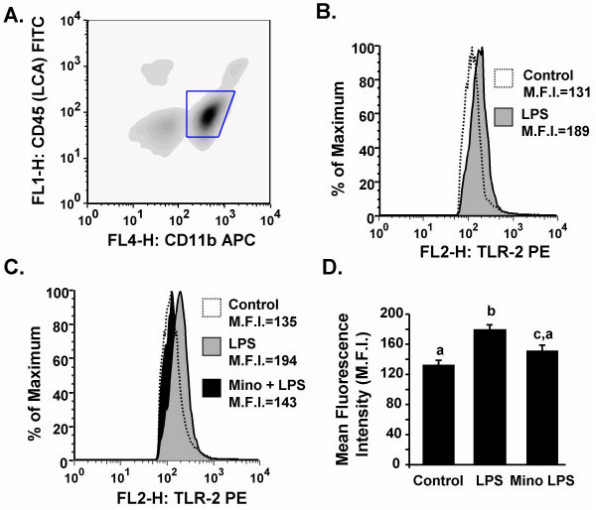
**LPS-induced TLR2 surface expression on microglia is reduced by minocycline**. Adult mice were injected i.p. with vehicle or minocycline for three consecutive days. On the third day mice were challenged with saline or LPS i.p., microglia and macrophages were collected 24 h later and TLR2 surface expression was determined. A) Representative bivariate density plot of stained cells. Macrophages were CD11b^+^/CD45^high ^and microglia were CD11b^+^/CD45^low^. B&C) Representative histograms of TLR2 expression on microglia following experimental treatments. C) Mean fluorescence intensity (M.F.I.) of TLR2 expression on microglia (CD11b^+^/CD45^low^) following experimental treatments. Bars represent the mean ± SEM (n = 6). Means with different letters (a, b, or c) are significantly different (*P *< 0.05) from each other.

Because minocycline attenuated LPS-induced cytokine secretion and TLR2 mRNA expression in BV-2 cells we next sought to determine if minocycline suppresses markers of microglial activation in the brain of mice. Mice were injected i.p. with vehicle or minocycline for 3 consecutive days then challenged with saline or LPS i.p. Markers of activation, TLR2 and MHC II, were determined on microglia collected 24 h later. The representative bivariate density plot in Fig. 2A shows that there were two populations of CD11b/CD45 positive cells and that more cells stained CD11b^+^/CD45^low ^(microglia) than CD11b^+^/CD45^high ^(CNS macrophages). ANOVA revealed that LPS injection increased TLR2 surface expression on microglia (F(1, 20) = 17.6, *P *< 0.004, Fig. 2B&D), but this induction was abrogated by minocycline pretreatment (Tendency for Mino × LPS interaction, F(1, 20) = 2.66, *P *= 0.10, Fig. 2C&D). It is important to note that because minocycline and saline controls did not differ in their TLR2 expression, these data were grouped together as the *Control *group (Fig. 2B&C). In addition, neither minocycline nor LPS treatment had a significant main effect on MHC class II surface expression on microglia (data not shown). These data indicate that minocycline attenuated LPS-induced TLR2 expression on microglia.

### Minocycline facilitates the recovery from LPS-induced sickness behavior

**Figure 3:**
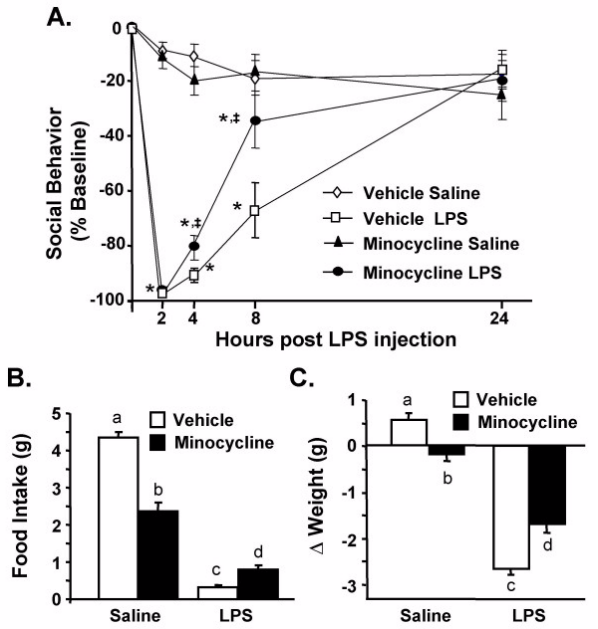
**Minocycline facilitates recovery from LPS-induced sickness behavior**. Mice were injected i.p. with vehicle or minocycline for three consecutive days. On the third day mice were challenged with saline or LPS i.p. A) Social exploratory behavior was measured before i.p. LPS injection and again 2, 4, 8, and 24 h later. Graph represents the mean ± SEM (n = 10). Means with * are significantly different (*P *< 0.05) from saline controls and means with ‡ are significantly different from *Vehicle LPS*. In the same experiment, B) body weight and C) food intake were measured before i.p. LPS injection and again 2, 4, 8, and 24 h later. Bars represent the mean ± SEM (n = 10). Means with different letters (a, b, c, or d) are significantly different (*P *< 0.05) from each other.

CNS macrophages and microglia produce inflammatory cytokines and secondary messengers that modulate behavioral responses. Therefore, we next investigated if minocycline reduced the sickness response associated with peripheral LPS injection. In this experiment, adult mice were treated with minocycline and LPS as described. Social exploratory behavior was measured before i.p. LPS injection and again 2, 4, 8, and 24 h later. Fig. 3A shows that LPS injection caused a reduction in social exploratory behavior (F(1,57) = 218, *P *< 0.001) that was time dependent (F(4,57) = 66.5, *P *< 0.001). Moreover, the LPS-associated reduction in social exploration was attenuated by minocycline (Mino × LPS interaction, F(1,57) = 7.5, *P *< 0.007). For example, at 8 h post LPS, social exploration was reduced by 35% in minocycline pretreated mice given LPS compared to a 67% reduction in vehicle pretreated mice given LPS (*P *< 0.001). While minocycline administration alone reduced food intake and body weight in control mice *(P *< 0.05, for each), it also protected against LPS-associated anorexia (Mino × LPS interaction, F(1, 60) = 70.0, *P *< 0.001, Fig. 3B) and weight loss (Mino × LPS interaction, F(1, 60) = 29.7, *P *< 0.001, Fig. 3C).

**Figure 4:**
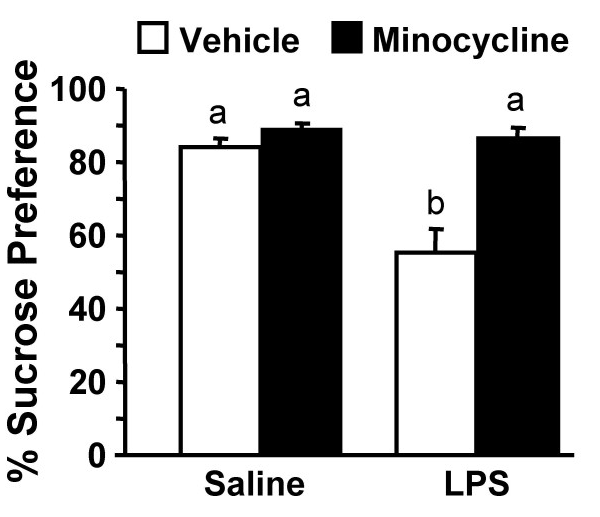
**LPS-associated anhedonia is blocked by minocycline**. Mice were injected i.p. with vehicle or minocycline for three consecutive days. On the third day mice were challenged with saline or LPS i.p. and sucrose preference was determined 24 to 39 h post LPS. Bars represent the mean ± SEM (n = 15). Means with different letters (a or b) are significantly different (*P *< 0.05) from each other.

Because sickness can also be associated with longer lasting changes in motivation [38], we next sought to determine if minocycline abrogated LPS-induced anhedonia [54,55]. In this experiment, mice were subjected to the same minocycline injection regimen and LPS challenge as above and sucrose preference was assessed 24–39 h post LPS injection. By 24 h post LPS injection, food and water intake returned to baseline and LPS treated mice still exhibited a marked reduction in sucrose preference from 24–39 h (F(1,59) = 14.3, *P *< 0.003). Moreover, this LPS-dependent reduction in sucrose preference was prevented by minocycline pretreatment (Mino × LPS interaction, F(1, 59) = 9.9, *P *< 0.004, Fig. 4). For example, minocycline pretreated mice injected with LPS maintained the same strong preference for sucrose as saline and minocycline controls (i.e., approximately 85% preference). These data can be interpreted to indicate that minocycline blocks anhedonia associated with peripheral LPS challenge.

### Minocycline reduces LPS-induced neuroinflammation

**Figure 5:**
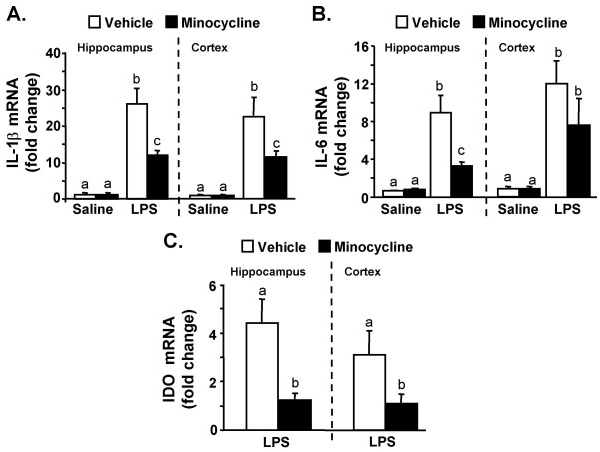
**Minocycline attenuates LPS-induced mRNA levels of IL-1β, IL-6, and IDO in the cortex and hippocampus of adult mice**. Mice were injected i.p. with vehicle or minocycline for three consecutive days. On the third day mice were challenged with either saline or LPS i.p and A) IL-1β, B) IL-6, and C) IDO mRNA levels were determined in the cortex and hippocampus collected 4 h later. Bars represent the mean ± SEM (n = 8). For each brain region, means with different letters (a, b, or c) are significantly different (*P *< 0.05) from each other.

Pro-inflammatory cytokines in the CNS are partially responsible for the behavioral symptoms of sickness (e.g., anorexia, social withdrawal, and anhedonia) [1]. Therefore, we investigated the degree to which minocycline reduces neuroinflammation (IL-1β, IL-6, and IDO) after peripheral injection of LPS. In this experiment, mice were subjected to the minocycline injection regimen and LPS challenge as above and cytokine mRNA levels were determined in the cortex and hippocampus 4 h after LPS injection. In mice pretreated with vehicle, LPS markedly increased IL-1β mRNA levels in the hippocampus (F(1,31) = 62, *P *< 0.0001) and cortex (F(1,31) = 17.25, *P *< 0.0003). The LPS-induced IL-1β mRNA expression was reduced in both brain regions in mice receiving minocycline prior to LPS injection: (hippocampus, F(1,31) = 9.63, *P *< 0.01) and cortex, F(1,31) = 7.23, *P *= 0.08, Fig. 5A). LPS caused a similar induction of IL-6 mRNA levels in the hippocampus (F(1,31) = 37.2, *P *< 0.001) and cortex (F(1,31) = 22.5, *P *< 0.001), but minocycline pretreatment only significantly attenuated LPS-induced IL-6 mRNA levels in the hippocampus (F(1,31) = 10.27, *P *< 0.004, Fig. 5B).

**Figure 6:**
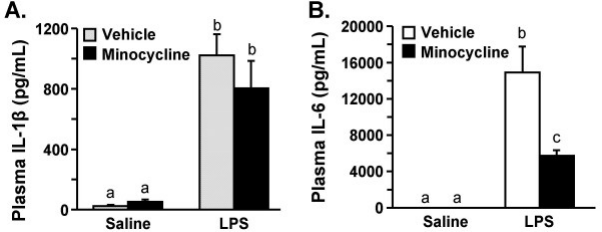
**Minocycline reduces LPS-induced IL-6, but not IL-1β, levels in the plasma**. Mice were injected i.p. with vehicle or minocycline for three consecutive days. On the third day mice were injected with saline or LPS i.p. IL-1β and IL-6 were determined in plasma collected 4 h later. There was no detectable IL-6 (n.d.) in the plasma of saline-treated mice. Bars represent the mean ± SEM (n = 8). Means with different letters (a, b, or c) are significantly different (*P *< 0.05) from each other.

IDO mRNA levels were determined from the same RNA pool. Fig. 6D shows that LPS injection increased IDO mRNA expression in the hippocampus (F(1,31) = 11.69, *P *< 0.002) and cortex (F(1,31) = 5.26, *P *< 0.03). This LPS-induced IDO mRNA expression was attenuated by minocycline in the hippocampus (F(1,31) = 11.69, *P *< 0.002) and cortex (F(1,31) = 5.26, *P *< 0.03). It is important to note that IDO mRNA was undetected in saline treated mice. Therefore, the fold IDO change was relative to the IDO mRNA levels in mice receiving minocycline prior to LPS.

### Minocycline reduces LPS-induced IL-6, but not IL-1β, in the plasma

Because cytokine signals can be relayed from the periphery to the brain by humoral pathways [56], plasma cytokine levels of IL-6 and IL-1β were determined 4 h post LPS injection. As expected, LPS injection caused a marked increase in plasma IL-1β (F(1,36) = 52.5, *P *< 0.001) and IL-6 levels (F(1,36) 34.01, *P *< 0.01). Minocycline pretreatment reduced LPS-induced IL-6 levels in the plasma (F(1,36) 6.68, *P *< 0.01) but had no significant main effect on LPS-induced IL-1β levels (Fig. 6).

### Minocycline attenuates LPS-induced exaggerated neuroinflammation in aged mice

**Figure 7:**
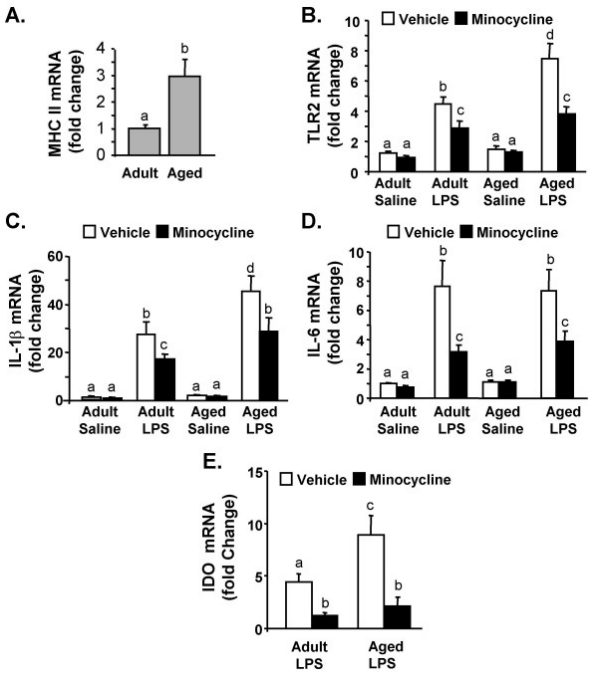
**Minocycline pretreatment attenuates LPS-induced mRNA levels of TLR2, IL-1β, IL-6, and IDO in the hippocampus of aged mice**. Adult and aged mice were injected i.p. with vehicle or minocycline for three consecutive days. On the third day mice were challenged with saline or LPS i.p. and A) MHC II, B) TLR2, C) IL-1β, D) IL-6, and E) IDO mRNA levels were determined from hippocampus collected 4 h later. Bars represent the mean ± SEM (n = 8). Means with different letters (a, b, c, or d) are significantly different (*P *< 0.05) from each other.

Aged BALB/c mice (22–24 m) have an exaggerated neuroinflammatory response to LPS injection [10,13,14]. Therefore, we next sought to determine if the heightened inflammatory response in the brain of aged mice was reduced by minocycline. In this experiment, adult and aged mice were subjected to the minocycline injection regimen and LPS challenge as above. As we have reported previously, MHC II mRNA expression was increased by age (*P *< 0.03, Fig. 7A)[13,14], but MHC II levels were unaffected by either LPS or minocycline treatment (not shown). Consistent with the data presented in Fig. 2, ANOVA revealed a significant main effect of LPS injection on TLR2 mRNA expression in the hippocampus (F(1,63) = 85.5, *P *< 0.001). Moreover, LPS caused a greater increase in TLR2 mRNA in the hippocampus of aged mice compared to adults (LPS × Age interaction, F(1,63) = 12.70, *P *< 0.01). Furthermore, minocycline pretreatment attenuated LPS-induced TLR2 mRNA levels in both adult and aged mice (Mino × LPS interaction, F(1,63) = 9.02, *P *< 0.004).

Parallel to the results for TLR2, LPS caused a greater increase in IL-1β and IDO mRNA levels in hippocampus of aged mice compared to adults (Age × LPS, F(1,60) = 8.64, *P *< 0.01 for IL-1β and F(1,60) = 4.0, *P *< 0.05 for IDO). Minocycline pretreatment attenuated LPS-induced mRNA levels of IL-1β (Mino × LPS, F(1,60) = 8.76, *P *< 0.01, Fig. 7C) and IDO (Mino × LPS, F(1,60) = 9.7, *P *< 0.003, Fig. 7D). While LPS induced higher IL-6 mRNA levels in the hippocampus of both adult and aged mice (F(1,59) = 44.5, *P *< 0.001), there was not an Age × LPS interaction. Minocycline pretreatment attenuated the LPS-induced increase in hippocampal IL-6 mRNA (Mino × LPS, F(1,59) = 5.4, *P *< 0.02, Fig. 7E). Taken together these data indicate that minocycline pretreatment was effective in attenuating the exaggerated neuroinflammation in aged mice.

## Discussion

In the elderly, systemic infection is associated with an increased frequency of behavioral and cognitive complications [57,58]. We have reported that stimulation of the peripheral immune system in older (20–24 m) BALB/c mice causes exaggerated neuroinflammation that is paralleled by prolonged sickness [13], impaired working memory [10], and depressive-like behaviors [15]. Therefore, it is important to understand the mechanisms that can modulate cytokine-mediated pathways in the brain. Here we show that minocycline treatment reduced LPS-induced TLR2 expression in BV-2 cells and on microglia isolated from adult mice. Moreover, we demonstrate that minocycline was effective in facilitating the recovery from LPS-induced sickness and preventing anhedonia in adult mice. Furthermore, we show that minocycline attenuated LPS-induced neuroinflammation in adults and normalized the exaggerated neuroinflammation in aged mice.

Our findings, using cell culture and animal experiments, support the notion that minocycline attenuates microglial activation and limits production of inflammatory mediators. For instance, minocycline pretreatment of BV-2 cultures decreased LPS-stimulated cytokine production in a dose dependent manner (Fig. 1A). In BV-2 cells, minocycline also attenuated mRNA expression of inflammatory genes including IL-6, IL-1β, MHC II, and TLR2 (Fig. 1D). These data are consistent with other studies using minocycline and LPS in BV-2 cells [44,59]. Based on these data we next investigated if microglial activation could be attenuated in the brain. Because LPS increases brain cytokine production we expected that MHC II expression would also be increased. Contrary to our predictions, neither MHC II mRNA levels (Fig. 7) in the brain nor MHC II surface expression on microglia (CD11b^+^/CD45^low^) (data not shown) were increased by LPS injection. In an EAE model, minocycline reduced microglial expression of MHC II [45], but one key difference from our study is that the induction of EAE pathology requires functional antigen presentation on MHC II [60]. It is postulated that microglia have several activation states that depend on the specific inflammatory stimulus [61]. Thus, in situations of transient peripheral innate immune stimulation, markers in the CNS such as Toll-Like receptors [6] may be indicative of microglia activation. In support of this premise, our data show that LPS injection increases TLR2 surface expression on microglia (CD11b^+^/CD45^low^), which is inhibited by minocycline pretreatment (Fig. 2). These data are consistent with other studies showing that central or peripheral LPS challenge increases TLR2 mRNA in the brain [6,14]. Taken together our findings can be interpreted to suggest that minocycline attenuates pathways associated with microglia activation following peripheral LPS challenge.

One of the important findings of this study was that reduction of neuroinflammation by minocycline was associated with facilitated recovery from LPS-induced sickness behavior. These results are akin to our previous work with the anti-oxidant, α-tocopherol [52], and an NFKB decoy inhibitor [62]. Consistent with our previous studies [52,53,62,63], reductions in neuroinflammatory cytokines (Fig. 5) did not prevent the induction of the LPS-induced sickness response (2–4 h), but rather facilitated the recovery from sickness (8–24 h) (Fig. 3A). Recovery may be a critical issue because brain cytokines and the corresponding physiological and behavioral responses are beneficial to the host [1]. The potential risk for a maladaptive response occurs when the normally transient neuroinflammatory response is amplified or protracted [64]. Therefore pharmacological agents, similar to minocycline, that attenuate neuroinflammatory responses, but do not completely inhibit them, may be important in preventing the development of more severe and long-lasting cognitive and behavioral complications.

The results of the sucrose preference experiments support the idea that limiting exposure to neuroinflammation decreases the duration of behavioral responses. For example, while minocycline did not inhibit cytokine expression (Fig. 5) or the induction of sickness (Fig. 3A), minocycline pretreatment completely reversed the reduction in sucrose preference (i.e., anhedonia) associated with LPS injection (Fig. 4). It is also important to mention that while LPS-associated sickness and anhedonia are interrelated, these behaviors can be differentiated from one another. For instance, reduced social exploration was evident 2–24 h post injection (Fig. 3A), but only decreased sucrose preference was exhibited 24 to 39 h later (Fig. 4). This separation between behaviors is consistent with other studies investigating sickness and longer-lasting changes in motivation [15,65,66].

IDO mediated TRP metabolism represents a potential connection between activation of CNS innate immune cells and longer lasting behavioral responses. IDO mediated TRP metabolism in the brain may affect behavior by impacting both serotonin and glutamate pathways [39]. We have reported that IDO induction and activity is amplified in the brain of aged mice and is associated with prolonged depressive-like behavior [15]. Here we show that IDO mRNA induction is blocked by minocycline in the brain of both adult and aged mice (Figs. 5&7). These data are consistent with a recent report showing a causal relationship between IDO activity and acute depressive effects in adult CD-1 mice. In this study, O'Connor et al. report that both 1-methyl tryptophan (a competitive inhibitor of IDO) and minocycline blocked IDO induction and prevented depressive-like immobility in the tail suspension and forced swimming tests [66]. Thus, in the present study, the minocycline blockade of IDO induction may explain the abrogation of LPS-induced anhedonia.

Another interesting finding was that while minocycline pretreatment in adult mice attenuated LPS-induced brain IL-1β at 4 h (Fig. 5), it had no effect on plasma IL-1β levels (Fig. 6). Because IL-1β signals can be relayed from the periphery to the brain by humoral pathways [5], these findings suggest that minocycline has anti-inflammatory properties within the brain. These data are consistent with related findings that minocycline readily crosses the blood brain barrier to elicit an anti-inflammatory effect [41-43]. With regard to IL-6, minocycline pretreatment attenuated both brain and plasma levels at 4 h post LPS injection. Because circulating IL-6 levels can be increased by CNS mediated pathways including activation of the hypothalamus-pituitary-adrenal (HPA) axis [67] and the sympathetic nervous system [68], the specific reduction in plasma IL-6 by minocycline may reflect the reduction in brain inflammation at 4 h (Fig. 5). In support of this notion, we and others have reported that i.c.v. injection of LPS or IL-1β increase plasma IL-6 levels, but not IL-1β levels [14,68,69].

The final critical finding of this study was that minocycline was effective in attenuating neuroinflammation independent of age. Consistent with other aging and neuroinflammation studies, our data show that LPS caused exaggerated neuroinflammation in aged mice compared to adults [10,13-15]. It is important to mention that while there was an age-related difference in MHC II expression in the hippocampus of saline treated mice (Fig. 7A) there was not an age-related difference in IL-1β and IL-6 mRNA levels. These data differ from a previous report using BALB/c mice showing an increase in IL-6 in older mice [70]. This may be because the mice used in the present study were approximately 4 months younger than the mice used previously. Nonetheless, microglia can be primed or reactive with increased MHC II expression, but do not necessarily produce inflammatory cytokines in this state [19]. The key results are that peripheral LPS injection causes a greater induction of TLR2, IL-1β, and IDO mRNA in the aged brain than in the adult brain and that minocycline pretreatment normalizes this age-related exaggerated neuroinflammation (Fig. 7). These findings are also important because an amplified neuroinflammatory response in the aged brain is a precursor to complications including deficits in working memory, memory consolidation, and depressive-like behavior [9,10,15]. Based on the biochemical and behavioral data obtained from this study, we predict that minocycline will abrogate the prolonged LPS-induced sickness [13] and depressive-like behavior exhibited by aged BALB/c mice [15]. We acknowledge, however, that future studies are necessary to test these predictions.

## Conclusion

The present study demonstrates that minocycline reduces LPS-induced microglial activation, CNS cytokine production, and behavioral symptoms of sickness (e.g., social withdrawal and anhedonia). These findings are potentially important because they indicate that minocycline can be used to mitigate cytokine expression in the brain and have a beneficial affect on behavioral responses. Taken together, these data support the idea that pharmacological strategies aimed at decreasing neuroinflammation associated with microglial activation are important for improving recovery from sickness and reducing the frequency of neurobehavioral complications.

## List of abbreviations

3-Hydroxy-L-Kynuriene (3HK), Allophycocyanin (APC), Analysis of variance (ANOVA), Central Nervous System (CNS), Dulbecco's Modified Eagle's Medium (DMEM), Dimethyl Sulfoxide (DMSO), Experimental Autoimmune Encephalomyelitis (EAE), Enzyme Linked Immmunosorbent Assay (ELISA), Fluorescein Isothiocyanate (FITC), Fetal Bovine Serum (FBS), Hank's Balanced Salt Solution (HBSS), Indoleamine 2, 3 dioxygenase (IDO), Intraperitoneal (i.p.), Intracerebroventricular (i.c.v.), Interleukin (IL), Kynurenine (KYN), Lipopolysaccharide (LPS), Major Histocompatibility Complex class II (MHC II), Mitogen Activated Protein Kinase (MAP-kinase), Nuclear factor kappa B (NFκB), N-methyl-D-aspartate (NMDA), R-Phycoerthrin (PE), Quinolinic acid (QUIN), Statistical Analysis Systems (SAS), Standard Error of the Mean (SEM), Toll-like Receptor-2 (TLR2), and Tryptophan (TRP).

## Competing interests

The authors of this manuscript declare that there are no actual or potential conflicts of interest. The authors affirm that there are no financial, personal or other relationships with other people or organizations that have inappropriately influenced or biased their research.

## Authors' contributions

CJH was involved in research experimentation, completion of statistical analysis, and writing of the manuscript. YH, AW, MH and JH assisted with experimentation and data analysis. MB and JFS contributed to the design of the experiments and assisted in editing the manuscript. JPG directed all aspects of this research project including the experimental design, research experimentation, completion of statistical analysis, and writing of the manuscript.
